# Valorization of Chicken Feet By-Product of the Poultry Industry: High Qualities of Gelatin and Biofilm from Extraction of Collagen

**DOI:** 10.3390/polym12030529

**Published:** 2020-03-02

**Authors:** José C. C. Santana, Roberta B. Gardim, Poliana F. Almeida, Giovanna B. Borini, Ada P. B. Quispe, Segundo A. V. Llanos, Jorge A. Heredia, Stella Zamuner, Felix M. C. Gamarra, Thiago M. B. Farias, Linda L. Ho, Fernando T. Berssaneti

**Affiliations:** 1 Department of Production Engineering, Polytechnic School of University of São Paulo, Av. Prof. Luciano Gualberto, 1380-Butantã, São Paulo 05508-010, Brazil; poliana.almeida@svc.ifmt.edu.br (P.F.A.); lindalee@usp.br (L.L.H.); fernando.berssaneti@usp.br (F.T.B.); 2 Industrial Engineering Post Graduation Program, Nine July University, Vergueiro Avenue, 235/249, Liberdade, São Paulo-SP 01504-000, Brazil; roberta.bgardim@hotmail.com (R.B.G.); giovanna_borini@hotmail.com (G.B.B.); stella.rz@uni9.pro.br (S.Z.); 3 Federal Institute of Mato Grosso, São Vicente Campus, São Vicente da Serra, 78106-000 MT, Brazil; 4 Chemical Engineering Department, National University of Pedro Ruiz Gallo. Calle Juan XXIII 391, Lambayeque 14013, Peru; abarturen@unprg.edu.pe (A.P.B.Q.); svasquezll@unprg.edu.pe (S.A.V.L.); 5 Business School, Universidad del Pacífico, Calle Sanchez Cerro 2141 Jesús Maria, Lima 11, Peru; ja.herediap@up.edu.pe; 6 University of Brasília, Gama Campus, St. Leste Projeção A—Gama Leste, Brasília-DF 72444-240, Brazil; 7 Federal University of São Paulo, Baixada Santista Campus, Santos, São Paulo 11015-020, Brazil; thiago.michel@unifesp.br

**Keywords:** chemical quality, sensorial quality, chicken feet, collagen, health care product, gelatin, biofilms

## Abstract

In this research, products with high quality were obtained from natural sources. The sensorial qualities, chemical characterization, and physical properties of gelatin extracted from chicken feet were compared with commercial gelatins. The extraction process was performed using acetic acid on a concentration ranging from 0.318% to 3.682%, processing time between 1.0 h and 8.4 h and extraction temperature between 43.3 °C and 76.8 °C. After the end of each assay, the yield was measured. Results showed that, under the best conditions, the collagen extraction yield was above 8%, and comprised 78.525 g/100 g of protein. Collagen analyzed by ICP-MS was composed of 99.44% of macro-minerals that are of great importance to human health. ATR-FTIR analysis showed that approximately 70.90% of the total protein from chicken feet is collagen, whereas, in commercial gelatin, only 30.31% is collagen. When comparing chicken gelatin with commercial gelatin, most sensory attributes were similar and chicken gelatin gained acceptance by more than 80% of the consumers. Additionally, the collagen films obtained from chicken feet and swine showed water absorption, odors, and texture characteristics similar to commercial material, such as latex and celofane. Consequently, due to its similarity to human skin, it is possible to apply it as a biocurative.

## 1. Introduction

The demand and requirements of consumers and the international regulations for food products is frequently inclined towards healthy products with improved quality. These demands include foods with high nutritional value (e.g., fiber, proteins, and micronutrients), and foods with excellent sensory aspects, fewer calories, and with low sugar, salt, fat, and other undesirable characteristics. This scenario has stimulated research exploring new sources of food and utilizing by-products or wastes. For these reasons, there is interest in these new food products called functional foods [1,2,3].

In this context, collagen and gelatin extracted from natural sources have been demonstrated in many applications as food additives, and have recently been explored in the pharmaceutical and medical fields. Usually, the most popular commercial product is gelatin from mammals (pigs and cattle), which is sometimes subjected to greater restrictions and skepticism among consumers, owing to socio-cultural and health concerns [3,4,5,6]. However, owing to its nutritional value, the demand for collagen and gelatin from porcine skin has been increasing progressively since 1990 [7,8]. In addition, studies have recently explored other natural sources for collagen and gelatin, including duck feet, fish, and poultry [8,9].

Thus, collagen and gelatin from natural sources like chicken feet merit special attention because, although chicken feet are considered waste material in various countries, they contain important nutrients and have essential health beneficial properties. In Brazil, chicken feet are regarded as waste from the poultry industry because Brazilian consumers do not have a habit of using chicken by-products like chicken feet and carcasses [3].

The world’s largest poultry producer is the USA. Brazil overtook China as the second largest producer of chicken meat, reaching more than 10 million tons in 2009 and reached more than 13 million in the last year [10]. However, this places a very low commercial price on by-products such as chicken feet, feathers, skin, and bones. If collagen is determined to be component of these by-products, the production of artificial skin, bio-curatives, gelatin, jelly, and spreads from chicken feet could become alternate economically viable options [3,11].

The main products of the poultry industry include live animals, eggs, and various cuts of meat. Some by-products are also commonly produced from the carcass, meat bran, bone meal, and feathers in order to add value to products [12,13,14,15]. In this regard, studies have been conducted to improve the quality and add value to poultry products, such as by introducing new techniques to prevent the microbial contamination of poultry meat [13,16,17,18,19,20,21]. Other methods include improvements in the animal’s diet to improve meat quality and carcass yield [22,23,24,25]. The use of ultraviolet, infrared, and gamma irradiation techniques and computer vision techniques have also been used for quality control of chicken breasts [12,21,26,27]. Ultimately, this has led to the development of new products such as jelly and chocolate spread from chicken feet collagen [3,11].

Most commercial gelatins are derived from mammalian sources, mainly pigs and cattle, but, due to sociocultural restrictions (Islam and Judaism) and the frequent occurrence of diseases such as bovine spongiform encephalopathy, which cause problems for human health, the use of products derived from mammals for processing of functional foods, cosmetics, and pharmaceuticals ends up being limited. Thus, studies have reported extraction of gelatin from chicken feet and have focused on the extraction method [8,28,29,30]. However, to the best of our knowledge, there are no previous studies that have investigated the quality of chicken feet collagen and gelatin as well and determined the presence of macronutrients and micronutrients in these products using analytical techniques. Thus, as chicken feet are waste from the meat industry, the extraction of their collagen and applying in the production of gelatins and biofilms will add value to this waste and will solve the socio-cultural problems associated with other by-products from the meat industry [3,4,5,11]. Therefore, this work focused on the extraction and chemical characterization of chicken feet collagen using some techniques such as spectroscopy-ATR-FTIR, ICP-MS, and others, and by comparing the sensorial qualities of the gelatin products with commercial gelatin.

## 2. Material and Methods

The reagents and equipment used in the preparation of biotherapeutic films were supplied by Nine July University (UNINOVE), acquired through accredited and qualified suppliers, while chicken tarsi were purchased from CEAGESP (Company of Warehouses and General Warehouses of São Paulo, São Paulo, Brazil). Collagen extraction from chicken tarsi was based on the work of [3].

### 2.1. Collagen Extraction Procedure

Warehouse Company and General Stores Company of Sao Paulo (CEAGESP), Brazil provided the chicken feet. Chicken feet were washed, the nails were removed, and the feet were again washed with cold water to remove any residual dirt or debris. Finally, the chicken feet were chopped, weighed, and placed in contact with acetic acid solution under the conditions used in the factorial design [3,4,5,31]. The assays followed a 2^3^ factorial design using acetic acid concentration, [*A*_c_] (%, m/m), processing time, *t* (h), and extraction temperature, *T* (°C), as factors that influence the extraction yield (y_exp_). The coding of the variables followed Equations (1a)–(1c):

(1a) $$x_{1}=\left[A_{c}\right]-2$$

(1b) $$\;x_{2}=\frac{t-5}{2}$$

(1c) $$x_{3}=\frac{T-60}{10}$$

The extraction systems comprised beakers to a total volume of 1.0 L, kept under constant agitation (magnetic stirring) at the planned temperature. Each factorial design assay used 100.0 g of chicken feet. The assays were performed in triplicate. Extraction yield was calculated based on the initial mass of chicken feet, using Equation (2):

(2) $$y_{exp}=100\times \frac{M_{collagen}}{M_{chicken\;feet}}$$

After execution of the tests, a model was obtained by the least squares method and its adjustment was verified using the analysis of variance (ANOVA). Optimization was performed using the response surface methodology (RSM) in Software Statistica 6.0 for Windows^®^, São Paulo, Brazil, based on the concept proposed by [32,33,34,35]. The extracted material was distributed in Petri dishes and placed in a vacuum oven at 55 °C for 12 h. The dry material was then ground to obtain a powder and was characterized according to the standards presented in Association of Official Analytical Chemists, AOAC [36].

### 2.2. Gelatin Preparation

The techniques used for good practices and food security were based on Resolution RDC n° 216/2004 of the National Health Surveillance Agency (ANVISA) on 15 September 2004 [32,35]. Requirements for analysis of chemical and microbiological composition in food are presented in this law. The research was approved by the ethics committee of the institution, and the group of evaluators was composed of undergraduate students from the UNINOVE chosen at random in university living environments, but with similar distribution between genders.

A commercial gelatin (cowhide) acquired from the Brazilian market was used as a comparative sample. In water heated to 60 °C, chicken feet collagen powder was mixed with sugar, dye, and artificial flavor, and then distributed in small plastic cups that were refrigerated for gel formation, following the preparation procedure of the manufacturer, Dr. Oetker^®^. Chicken gelatin powder, protein, sugar content, colorants, and other compositional components of the gelatin formulations [11] are shown in Table 1, for commercial gelatin as well. The flavors used in the gelatin formulations were grape and pineapple because to these flavors are the most consumed in Brazil.

The acceptability of gelatin samples was assessed using sensory affective tests by comparing with the sensorial qualities of a commercial gelatin. Twenty-five milliliters of gelatin samples was served to 50 consumers in codified plastic cups covered with a thin layer of plastic film, using a monadic presentation and a 9-point hedonic scale. The consumers also registered their consumption intent for each sample (yes or no). Sensorial characteristics such as appearance, aroma, flavor, texture, and overall aspects of the gelatins were evaluated.

The form used to translate the sensorial responses of consumers to a numeric value in a hedonic scale was as follows: 1—I disliked extremely, 2—I disliked very much, 3—I disliked moderately, 4—I disliked slightly, 5—I perceived no difference (neither like nor dislike), 6—I liked slightly, 7—I liked moderately, 8—I liked very much, and 9—I liked extremely [32,37,38,39]. Based on the frequency of responses, sensorial data were compared using Student’s *t*-test according to Equations (3) and (4) [38,40,41]. Gelatins of the same flavors were compared in pairs on the attributes: appearance, aroma, flavor and texture:

(3) $$S_{G}=\sqrt{\frac{\sum_{i=1}^{n_{1}}(x_{i}-\bar{x_{1}}{)}^{2}+\sum_{j=1}^{n_{2}}{\left(x_{j}-\bar{x_{2}}\right)}^{2}+\sum_{k=1}^{n_{3}}{\left(x_{k}-\overset{{}_{}}{x_{3}}\right)}^{2}}{N_{1}+N_{2}+N_{3}+\ldots -N_{t}}}$$

(4) $$t=\frac{\overset{{}_{}}{x_{1}}-\bar{x_{2}}}{S_{G}}$$

where *N*_1_, *N*_2_, *N*_3_, and *N_t_* are the sample numbers of each test.

### 2.3. Determination of Percent Composition

(a) Moisture: The moisture content of gelatin and chicken feet was determined by drying at 105 °C for 8 h, according to the standard method of the *Association of Official Analytical Chemists*, (A.O.A.C.) [36] and as described by [31] and [42]. The results are expressed as the percent weight loss during the drying process.
(b) Protein: The protein content in gelatin and chicken feet was determined by the Kjeldahl method [36]. The correction factor used was 5.55 for gelatin and 6.25 for chicken feet.
(c) Lipids: Determination of the lipid contents in gelatin and chicken feet samples was performed by the Soxhlet method. This uses direct extraction of fat using an organic solvent (ether). After 4 h, the ether was evaporated in an oven at 100 °C for 1 h; the residue was then cooled in a desiccator to measure the mass of the extracted oil [36,43,44].
(d) Ash: The total ash content of previously dried samples was determined by calcining at 500–600 °C for 4 h, following the methodology described by [43] and by A.O.A.C. [36].

### 2.4. Determination of Mineral Contents by the ICP-MS Technique

Gelatin mineral analysis was determined by inductively coupled mass spectroscopy (ICP-MS) following the method from [45,46]. Samples with diameter smaller than 0.08 m were homogenized (ultrasound strainer) and digested in a mixture of 5:2 nitric and hydrofluoric acid in a microwave oven (100 plus DGT model—Provecto Analytic) and analyzed in a quadrupole ICP-MS system (PerkinElmer ELAN 6100).

The equipment was operated under standard conditions with cell collision technology (CCT); using a collision cell with 8% (*v/v*) He in H_2_, on an automatic mode. The configuration of the instrument uses a mixed flame torch and Peltier inlet with a 1.5 mm quartz nozzle and an increased sensitivity with an Xi cone interface (plasma display). A parallel nebulizer (Miramist Burgener) was used for high solid content. To improve precision, an aspiration rate of less than 0.4 mL/min was selected. The elements determined were: Mn, Fe, Al, K, Ca, Ti, Mg, Na, Cr, V, Ni, Zn, Pb, Li, Cu, La, Ce, Th, U, Sr, and Be. Previously, calibration curves for each analyzed element were prepared with a robust ICP-MS analytical setting and gravimetric standard solutions from the dilution of each derived element stock solution [47,48].

### 2.5. FTIR Procedure

FTIR spectra of collagen samples were recorded using a horizontal ATR, through a Nicolet iS5 FTIR spectrometer equipped with an ATR/iD3 with an argon horizontal cell (Thermo Fisher Scientific^®^, EUA) at 16 °C. The spectra in the range of 400–4000 cm^−1^ were rationed and the automatic signals gained were collected in 32 scans at a resolution of 4 cm^−1^ against a background spectrum recorded from a clean empty cell at 16 °C [41,45,49,50,51].

### 2.6. Determination of Gel Strength

For this, a texture analyzer TA-XT2, Stable Micro System (Surrey, UK) was used for determination of gel strength. The gelatins prepared at different concentrations were conditioned and cooled at 25 °C, and poured in standard bloom jars. According with [52], the common gelatin concentration used to analyze gel strength of commercial gelatin in the gelatin industry is 6.67% (*w/w*); this value was used to prepare the commercial gelatin sample (cowhide). It was then refrigerated at 5–7 °C for 16 h prior to gel strength measurement. The operating conditions used were a cross-head speed of 0.5 mm/s, a load cell of 5 kg, and a flat bottom plunger of 0.5 cm in diameter. The bloom value (g) is obtained after the plunger penetrates into the gel to a depth of 4 mm. At this depth, the maximum force reading was obtained and translated as the bloom strength of the gel [53].

### 2.7. Chicken Collagen Film Production

The research works [4,54] were considered for the production of the collagen films from chicken. Consequently, the biofilms were produced in different types of plasticizers (propylene glycol and glycerine), as formulated in Table 2.

For the formulation, 10 g of collagen from chicken were weighed and stored. After that, 0.1 g of methylparaben and 5 g of glycerin (Formulation I) or propylene glycol (Formulation II) were weighed, and were transferred for a glass beaker. Seventy milliliters of distilled water were immediately added and homogenized, followed by a water bath until the solubilization of methylparaben was completed. Collagen from chicken was added posteriorly to homogenize until fully solubilized. The solution was filtered to remove impurities present. The pH range verified should be between 5 and 6. The volume of the solution was completed with distilled water until 100 mL and fractionated in acrylic petri dishes, followed by infrared drying at 70 °C.

### 2.8. Production of Biotherapeutic Collagen Films from Swine

The production of biotherapeutic collagen films from swine was in accordance with the research by [4,54]. For this purpose, 150 bloom of gelatin (gel forming capacity) will be used in different types of plasticizers as shown in Table 3.

Ten grams of collagen from chicken were weighed and stored. Immediately, 0.1 g methylparaben and 5 g glycerin (formulation III) or propylene glycol (formulation VI) were weighed, and transferred to a glass beaker, with approximately 70 mL of distilled water added, homogenized and followed by being put in a water bath until complete solubilization of methylparaben. After that, collagen from chicken was added to homogenize until complete solubilization. The solution was filtered to remove impurities present. The pH was remained between 5 and 6. The volume of the solution was completed with distilled water up to 100 mL and fractionated in acrylic petri dishes following the infrared dryer at 70 °C. After the formulation of films, these were characterized.

#### 2.8.1. Swelling Index Determination

The swelling test allows the prospect of degradation that is related to the degree of hydration of the system to be checked and determined in advance. This test is important to verify if the studied material presents structural stability during the period necessary for the formation of the new regeneration tissue. Free films of collagen from chicken feet were cut into 2.5 × 2 cm pieces and placed in glass petri dishes and left in the desiccator with silica gel for 24 h. After the elapsed time, they were removed from the desiccator and weighed, where these values were adopted as the weight of an initial zero time (*Ws*). Free films were left in 20 mL of 0.9% NaCl solution according to the established times: 1, 10, 30, and 60 min. At the end of each stipulated time, swollen films were weighed and their values recorded (*Wi*). Its calculation is done using Equation (5) [55,56]. The swelling index (*Si*) was determined in triplicate:

(5) $$Si(\%)=100\times (\frac{Wi-Ws}{Ws})$$

where:

- *Wi* represents the membrane mass after swelling at times 1, 10, 30, and 60 min;
- *Ws* represents the dry membrane mass at time zero.

The data were collected in the swelling test (Ii%), and a comparative graph was generated for each sample.

#### 2.8.2. Determination of Water Vapor Transmission (*WVT*)

The objective of the *WVT* test was to verify the permeabiization of biotherapeutic collagen films from chicken feet. *WVT* tests the biofilms were made in triplicate with propylene glycol and glycerin, and according to method B E96-66 from *American Society for Testing Materials/EUA* (ASTM) [56].

The biotherapeutic films were placed in glass Petri dishes containing 40% saturated NaCl solution per 72 h into desiccator. After the established time, they were prepared properly for the water vapor transmission rate (*WVT*) tests. In each cup (film of 10 cm area is weighed), 10 mL water was added. Then, the films to be investigated were individually fixed to the edge of the clip-film domes. The kits (cup + distiller water + film) were weighed (time zero) and placed in a desiccated silica gel desiccator. Samples were weighed at 24 h, 48 h, 72 h, and 96 h. For each interval, the values were recorded to calculate the water vapor transmission rate of these films. In addition, for the samples preparation, a control kit was prepared, which served as a comparative basis. *WVT* was calculated using Equation (6) [55]:

(6) $$TVW\left(\frac{g}{hcm^{2}}\right)=24\cdot g\cdot t\cdot a$$

where:

- “*g*” is mass loss,
- “*t*” the time in hour,
- “*a*” area of film (10 cm^2^).

The values obtained were shown in Figure 10 with the time intervals.

Additionally, the swelling index test and the water vapor transmission test were made in the collagen films developed from swine (frequently more used), in order to compare with the collagen films from chicken feet.

## 3. Results and Discussion

### 3.1. Extraction and Optimization of the Chicken Collagen Process

Table 4 shows the factorial design used in this experiment and the results of the extractions of collagen (*Y*), in the experimental setting (*Y*_exp_) and those calculated by the model (*Y*_calc_), according to each assay performed. It is noted that the extraction varied from 1.7% to 8.5% of collagen in the initial mass of the chicken feet. After execution of each assay, the collagen powder was obtained after drying at 50 °C for 12 h in a vacuum drier.

The yields found in this work are similar to those reported by Lim et al. [57], which ranged from 1.72% to 5.33% depending on the extraction method used. Hao et al. [58] in a study with sturgeon skin pretreated with Ca(OH)_2_ obtained a gelatin yield of 2.40% to 3.52%. Jamilah and Hervinder [59] reported gelatin yields of 5.39% and 7.81% upon collagen extraction from red and black tilapia, respectively. Chew et al. [60] found a gelatin yield of 7.25% from the fin after the extraction process. Therefore, the results presented in trials 5 and 6 were greater than all the results reported previously. However, in a study on ray skin (*Raja kenojei*) with 6 h-extraction, Ref. [61] identified a gelatin yield of 17.48% and Ref. [54] obtained a gelatin yield of 12% during collagen extraction from giant squid.

The analysis of variance was employed for determining significant variables, as shown in Table 5. The regression equations were submitted to the *F*-test for the coefficient of determination *R***^2^** and explaining the variances at 95% of the confidence level. According to [31,32,33,34,35], the first *F*-test (*F_calc_*/*F_tab_*) must be more than one for it to be significant, the second *F*-test must be less than 1 for it to be predictive, and *R^2^* explaining the variance must be next at 1.0 and 100, respectively. Table 5 lists the significant parameters and statistical test results of the models.

The calculated value for the *F*_1_ test was 22.947, being 4.5 times larger than the tabulated *F*_1_ (4.070), while the calculated *F*_2_ was 1.950, being 10 times smaller than the tabulated *F*_2_ (19.160); this indicates that the model is statistically significant and is adjusted to the experimental data. Another parameter that corroborated for the analysis was the coefficient of determination (*R*^2^), which was equal to 0.9806 and is close to 1.0, as indicated by [31,32,33,34,35]. In this manner, it can be affirmed that the model is adjusted and can be used to predict the value of the yield of collagen extraction from chicken feet, for the conditions presented in this work.

Equation (7) is the best fitting model to predict the extraction yield (*y*) value on the influence of acetic acid (*x*_1_), processing time (*x*_2_), and temperature (*x*_3_):

(7) $$\begin{array}{lll} y & = & 6.0421+0.9531x_{1}-1.1271x_{2}+1.9282x_{3}-0.0839x_{1}^{2}-0.7692x_{2}^{2}+0.1580x_{3}^{3}\ldots +\\ & & \ldots -0.7732x_{3}^{3}+0.7947x_{1}x_{2}-0.1750x_{1}x_{3}-0.6192x_{2}x_{3}+0.1172x_{1}x_{2}x_{3} \end{array}$$

**Figure 1:**
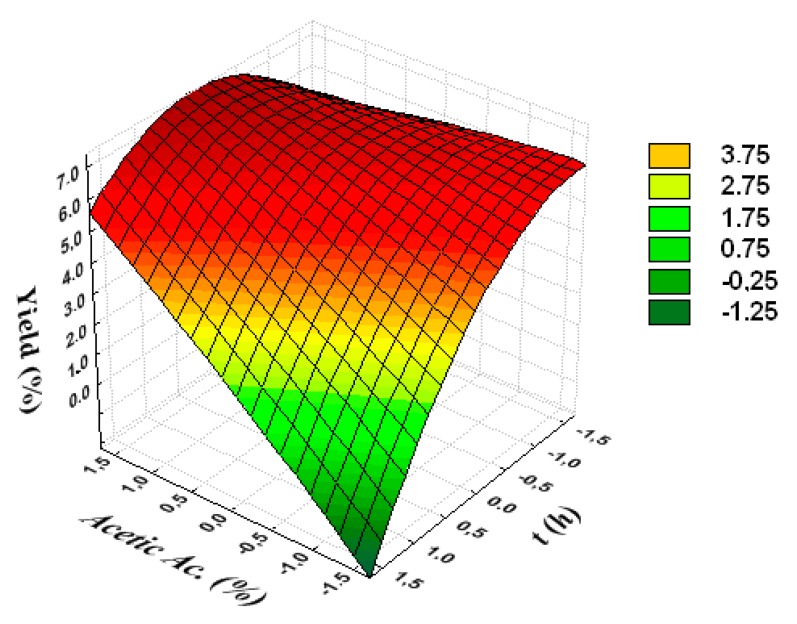
Response surface to show the mutual influence of acetic acid concentration and processing time on extraction yield.

**Figure 2:**
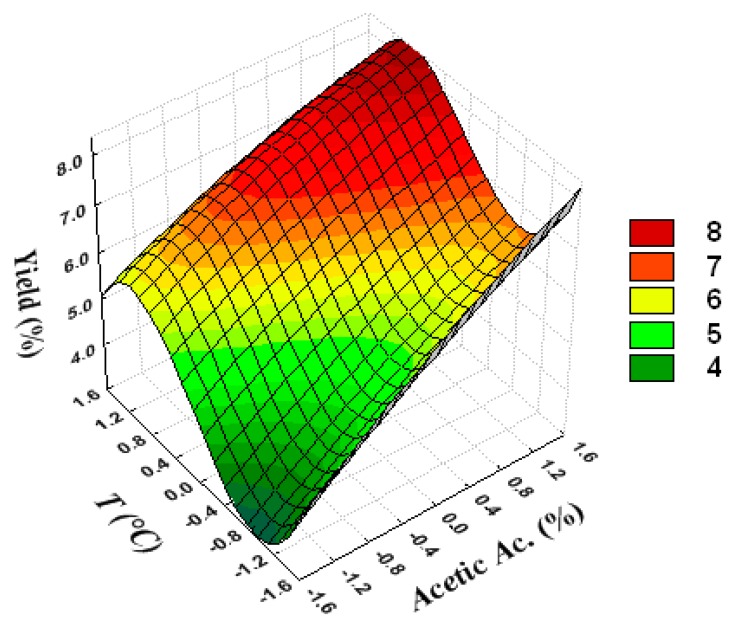
Response surface to show the mutual influence of temperature and acetic acid concentration on extraction yield.

**Figure 3:**
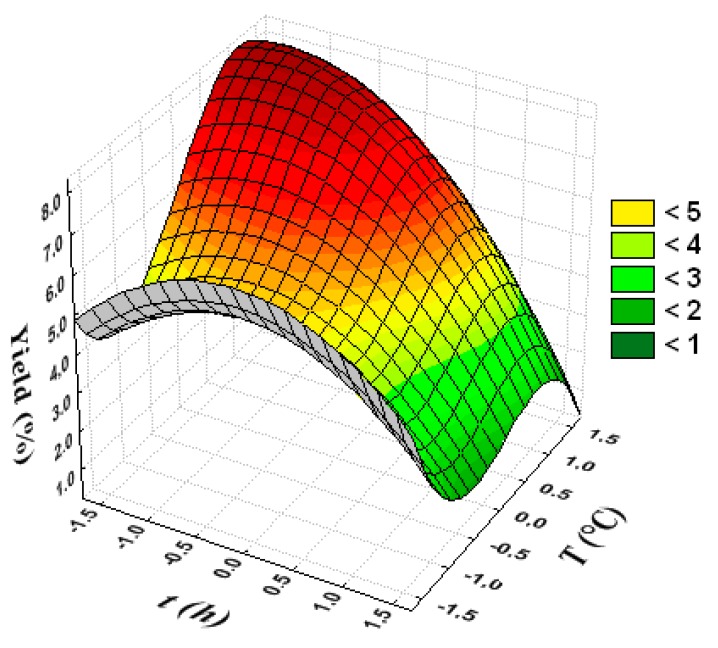
Response surface to show the mutual influence of processing time and temperature on extraction yield.

Figure 1, Figure 2 and Figure 3 are the response surfaces obtained in the Statistica 6.0^®^ software and are used in the optimization of collagen extraction from chicken feet. As shown in Figure 3, the highest yields are found when operated with the highest concentrations of acetic acid and with the shortest processing times.

Figure 2 shows that, if the temperature and acetic acid concentration are at their highest values, the yield is at a maximum.

Figure 3 shows that, in the shortest processing times and the highest temperature values, the yield tends to reach the maximum value. To find the best conditions, one must observe the level curves (lines) on the response surface, which tend to the highest values of extraction yield (in red).

Thus, it can be concluded that, by using acetic acid concentrations between 3.000% and 3.682%, operating times between 1 h and 3 h, and temperatures between 70 °C and 76.82 °C yielding greater than 8.0% collagen can be obtained.

### 3.2. Chemical and Bloom Analysis

Table 6 shows the chemical composition of chicken feet used for the extraction of collagen, with respect to the protein, lipid, ash, and water content. The powdered collagen composition was 9.7 g, 4.8 g, 6.9 g, and 78.5 g/100 g for moisture, ash, lipids, and proteins, respectively. Cliché et al. [62] also presented the composition of chicken feet, which had the following crude protein, ash, fat, and moisture content values of 17.42%, 12.04%, 5.98%, and 62.05%, respectively, which is similar to that found in our work.

The results of collagen gel strength from chicken feet are presented in Table 7. Upon comparing the gel strength of chicken feet gelatin to commercial gelatin at 6.67% collagen, this chicken feet gelatin has roughly 45% greater gel strength than that of commercial gelatin.

**Figure 4:**
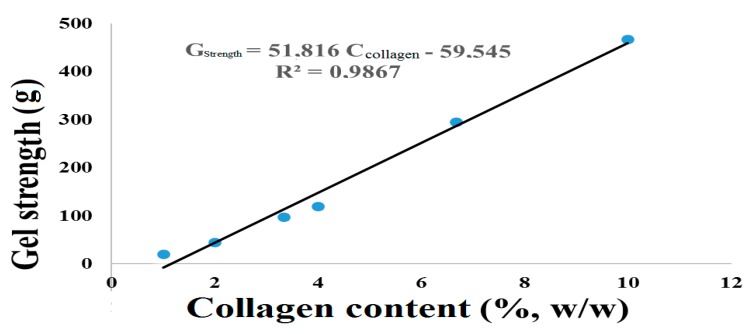
Variation of gel strength with the collagen content in gelatin.

Consequently, Figure 4 shows the variation in gel strength with the composition of the collagen in the gelatin. As expected, gel strength increases with the collagen composition in gelatin and, in this case, showed a linear increase. Using a linear equation obtained from Table 7 data, it was observed that, to obtain a gel strength similar to that of commercial gelatin, one requires only 5% collagen concentration from chicken feet gelatin. This equates to a reduction of about 24% (*w/w*) in the final gelatin composition, and thus makes chicken feet gelatin more economically feasible than commercial gelatin.

In a study on fish gelatin extraction with or without treatment by transglutaminase enzymes in a hydrolysis process, Norziah et al. [53] produced gelatin with low gel strength, varying between 70 g and 100 g, in addition to a commercial gelatin from halal bovine with a gel strength of 336.2 g. All gelatins presented in this study had low gel strength; however, in the same work, the authors cite a commercial fish gelatin with gel strength (435.9 g) approaching that found in this study. Thus, it is possible to suggest that the gel strength of chicken feet gelatin found in this work is superior or equal to that reported in previous studies.

### 3.3. Collagen Composition from Analysis of the FTIR

Recently, Ref. [27] used infrared spectroscopy to control the quality of intact chicken breast fillets by predicting the major component scores of qualities. In addition, Ref. [41] reports that this technique can be used to monitor the shelf life of products and the origin of agricultural products. Thus, in order to contribute to the quality control of chicken products, we used the FTIR technique in chicken jelly and commercial gelatin samples to verify the collagen content of both.

**Figure 5:**
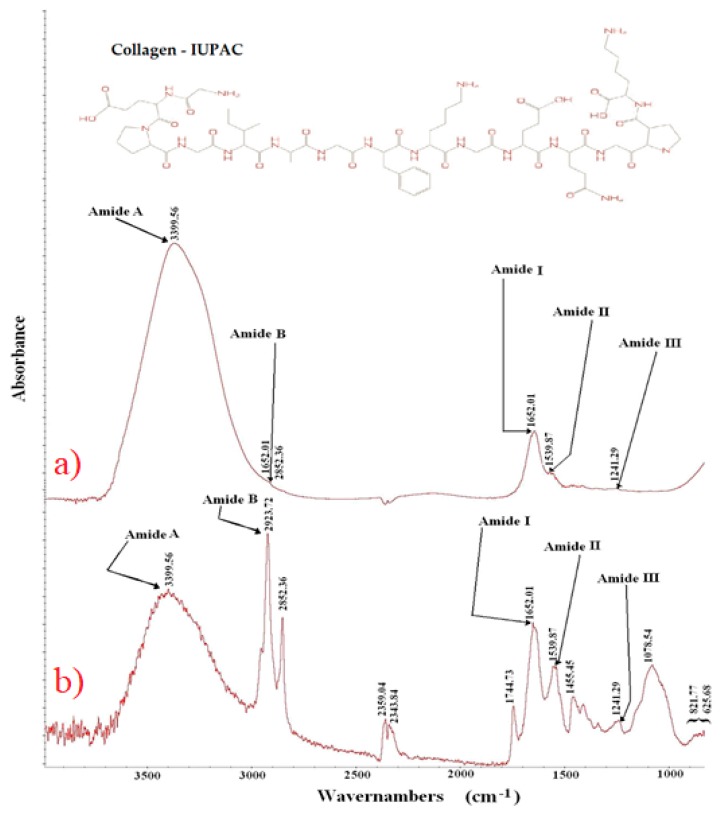
FTIR spectra of gelatins. (**a**) collagen from chicken feet, (**b**) collagen from commercial gelatin.

Figure 5a shows the FTIR spectra of collagen extracted from chicken feet on a thermal bath of 4% acetic acid solution at 60 °C for 4 h and Figure 5b shows the FTIR spectra of collagen from commercial gelatin. Each spectrum is an average of 32 scans of three batches on the optimal extraction conditions presented in the optimization section. FTIR spectra of the collagen extracted from chicken feet showed major peaks in the amide region. Specifically, chicken collagen showed a vibration peak at the wave numbers of 1652.01 cm^−1^ for amide I, of 1539.87 cm^−1^ for amide II, of 1241.29 cm^−1^ for amide III, of 2923.72 cm^−1^ for amide B, and of 3399.56 cm^−1^ for amide A. The FTIR spectra of commercial gelatin showed Amide II at 1556.53 cm^−1^, amide I at 1651.32 cm^−1^, Amide B at 2921.49 cm^−1^, and amide A in a range of 3391.84–3467.09 cm^−1^, and did not show any amide III peaks. However, a high protein content of low molecular weight has been found [51,63].

The low molecular weight peptides formed during extraction for long times were more likely to form covalent cross-links during the freeze-drying process [50,64,65,66]. This affected the collagen content from commercial gelatin and reduced one of the main qualities expected in gelatins. The process used in this work did not have the same effect on chicken gelatin. Results showed that collagen composition from chicken feet was greater than 70.90% while that from cowhide gelatin (commercial) was only 35%.

### 3.4. Mineral Composition

The chemical composition of ash was determined by mass spectroscopy (ICP-MS) and the results are reported in Table 8. Macro-minerals (Na, K, Ca, Mg, P, and S) that are of great importance to health human are presented and make up most of the elements (99.44% ash content) in the collagen composition.

As seen in Table 8, there are high levels of Na, at about 6 mg/g, resulting in 28.546 mg of Na per 100 g of powdered collagen. In the descending of quantity in 100 g of powdered collagen, Na is followed by Mg, Ca, S, and K, with contents of 8.817 mg, 8.136 mg, 7.236 mg, and 4.105 mg, respectively. Phosphorus is an important element for energy changes in cells via adenosine triphosphate transfer/adenosine diphosphate (ATP/ADP), and shows a significant amount in collagen from chicken feet.

Other important micro-minerals to human health such as Cu, Fe, and Mn are present in trace amounts (0.04% of ash). Similarly, the presence of toxic elements such as Pb, Ba, Al, Li, and Be is in trace amounts, which does not compromise this food (0.22% of ash content) [32,67], and the elements are all derived from chicken feet.

However, the commercial (powder) gelatin used in this work as a comparison has 108 mg of sodium per 5.7 g of powder, which is equivalent to 1847 mg per 100 g. Thus, the collagen obtained in this work presents low sodium content, which is in conformity with the new Brazilian laws that oblige companies to reduce the levels of sodium in their food products.

Using mass spectroscopy, Haug et al. [5] have found about 5.08 mg, 0.29 mg, 0.02 mg, and 7.13 mg of Na, Ca, K, and other heavy metals in each 1 g of fish gelatin. These values are 43.50% of the total ash content. Their macro-mineral percentage is lower than that of chicken feet gelatin, indicating that our gelatin is nutritionally superior to fish gelatin with respect to minerals.

Under the conditions used in this work, the quality of collagen powder allows production of gelatins that can be used to produce various cosmetics, facial masks, or even artificial tissues for wound healing in humans [68]. Thus, these products can be more easily marketed because of a good representation of chemical, nutritional, and sensorial qualities in their labels. This can possibly bring overall health improvement to humans, and being superior to commercial products, can facilitate greater acceptance by consumers [39].

### 3.5. Sensorial Qualities

Table 9 shows the sensorial comparison between chicken and commercial gelatin for two flavors. The table show that the consumer acceptances were higher than 80% for all samples tested in this study. The Student’s *t*-test showed no significant differences between the pineapple flavored gelatins for all sensorial qualities studied. However, for grape-flavored gelatins differences were observed for aroma, flavor, and overall vision; in all cases, commercial gelatins presented higher means than chicken collagen gelatins, mainly with the grape-flavored gelatin. However, since the values attributed to chicken collagen gelatin presented an average between 6.3 and 7.7 on a hedonic scale, they can be considered as accepted by consumers, of which 83% confirmed that they would consume this product.

The high values of commercial gelatin may be because of the high levels of sugar in its composition. The highest averages were obtained by commercial gelatin because of 72% sugar in its composition. Chicken gelatin had lower levels of sugar (28%) and high collagen content (71%), which gives it greater value with respect to health and nutrition for the consumers compared to commercial gelatin. Additionally, it can be more readily consumed by people with diabetes, owing to its low sugar content. Chicken gelatin may thus be considered as the best gelatin, since its sensory qualities were the best as assessed by consumers, with an acceptance average varying between 6.3 and 7.5 times on the hedonic scale for all sensorial attributes.

**Figure 6:**
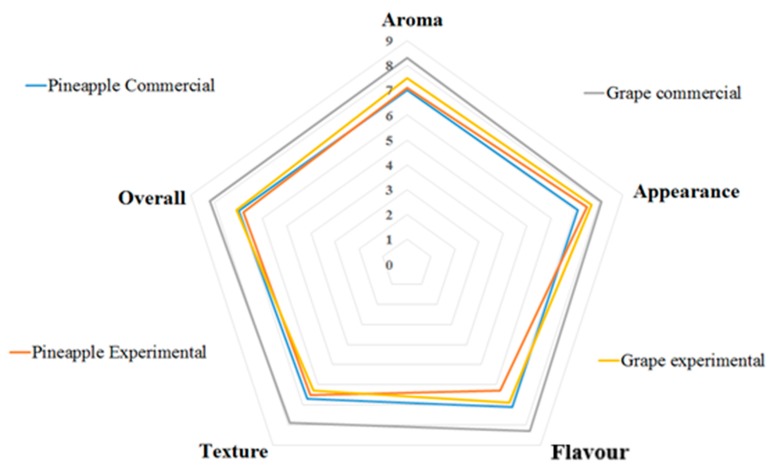
Response to sensorial qualities of gelatins.

Figure 6 shows that the majority of gelatins presented similar values for each attribute, which were close to seven points in a hedonic scale, thus indicating the similarity between chicken collagen (experimental) and commercial gelatins. However, grape-flavored commercial gelatin was significantly different among the others, since its average was above eight points on the hedonic scale

All attributes of chicken collagen gelatin were satisfactorily evaluated by consumers, since their average is close to seven points on the hedonic scale, indicating that this product has an excellent sensorial quality.

### 3.6. Biotherapeutic Collagen Films Properties

Table 10 shows that some organoleptic properties collected of biotherapeutic collagen from chicken feet. The films were completed with propylene glycol, and films made with glycerine showed pH, color, and odor, similar characteristics except the texture.

**Figure 7:**
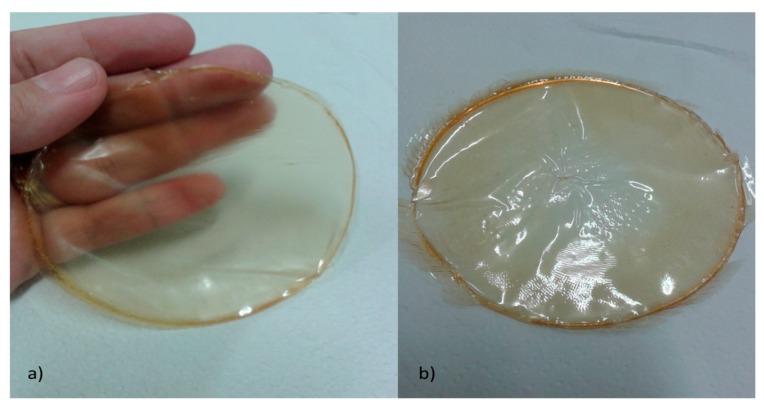
Film sample obtained from collagen of chicken feet with: (**a**) glycerin and (**b**) propylene glycol.

The biotherapeutic collagen film sample as shown in Figure 7. From this, it can be observed that films A (prepared with glycerin) are softer and than film B (prepared with propylene glycol). The texture found in Figure 7A showed similarities with texture of the latex used to make disposable gloves and condoms. However, the biotherapeutic collagen films prepared with propylene glycol (see Figure 7B) proved to be more rustic and rougher, having a similar appearance with cellophane material.

**Figure 8:**
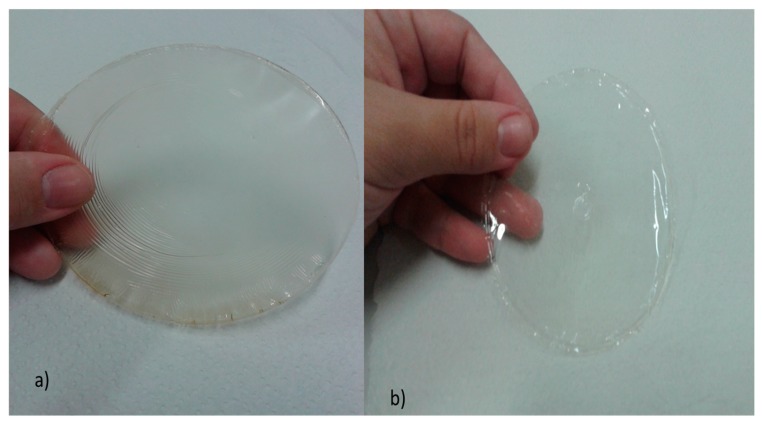
Films sample obtained from collagen of swine skin with: (**a**) glycerin and (**b**) propylene glycol.

Additionally, for comparative interpretation, biotherapeutic collagen films from swine were produced using one sample with propylene glycol and another sample with glycerin, as are shown in Figure 8. From this, it can be observed that the results were similar with those films produced from chicken feet, but the films from swine were odorless and lighter in color. In addition, comparative organoleptic characteristics of biotherapeutic collagen films from swine are shown in Table 11. 

### 3.7. Quality of Biotherapeutic Collagen Films from Chicken and Swine

**Figure 9:**
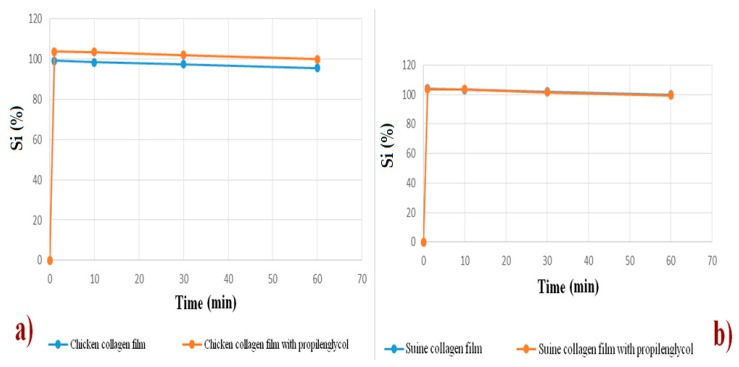
Polymer degradation process, based in swelling index curves. (**a**) chicken collagen samples and (**b**) swine collagen samples.

Swelling test allows checking and determining, in advance, the prospect of degradation, which is related to the degree of hydration of the system. These tests are important to verify the qualities and if the material has structural stability during the period necessary for the formation of the new regeneration tissue [52]. Right after 1 min, both films’ formulations were swollen to 99% of weight, and this remained unchanged until 60 min. This showed that there was a low degradation of the films. Films swell very fast. Comparing Figure 9a,b, it is noted that there is no difference between the collagen films of chicken feet and that of pig collagen. The swelling of the films is explained by its water absorption power, which is determined by the presence of hydrophilic groups and the high degree of crosslinking of the collagen microspheres [4,5].

**Figure 10:**
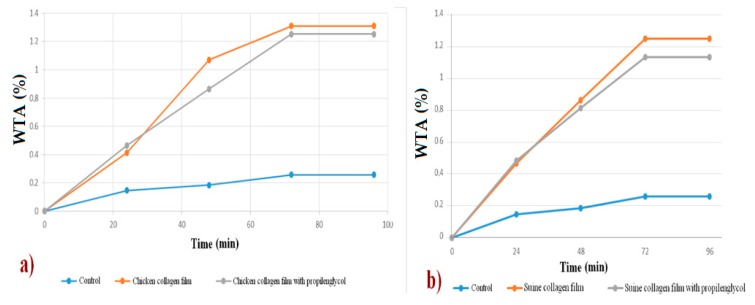
Water vapor transmission rate (*WVT*) test for films sample obtained from (**a**) chicken collagen samples and (**b**) swine collagen.

Water vapor transmission rate (*WVT*) tests were performed on biofilms produced from collagen of chicken feet and swine collagen, both with the plasticizers: glycerin and propylene glycol. Results are shown in Figure 10a,b. Regardless of the type of collagen and plasticizer used, all biofilms have similar results, with a satisfactory percentage of water vapor transmission, which allows its use as a biotherapeutic, since both allow similar transpiration to human skin.

## 4. Conclusions

In this work, the best extraction condition for collagen from chicken feet was found at 3.000%–3.682% acetic acid concentration, with variation of time between 1–3 h, and variation of temperature between 70 °C–76.82 °C. The gelatin analyzed by ICP-MS was composed of 99.44% Na, Ca, K, Mg, P, and S, which are macro-minerals of great importance to human health. Furthermore, FTIR characterization has shown that the collagen composition of chicken feet gelatins is two times greater than that of commercial cowhide gelatin. Therefore, the gelatin obtained is nutritionally richer than the marketed gelatin. In addition, the sensorial qualities of this gelatin were similar to commercial gelatin, and more than 80% of surveyed panelists would consume the chicken gelatin. In addition, the collagen biofilms were obtained from chicken feet using propylene glycol, and glycerol as plasticizers showed satisfactory results for swelling index (with 99% absorption capacity) and *WVT* test (without significant change), and had similar behavior to collagen films made from swine when they were compared. In addition, the collagen films made from chicken feet displayed texture characteristics similar to the appearance of latex and celofane commercial products. Additionally, these collagen films had various similar characteristics to the collagen film made from swine. However, more complementary research is necessary to validate their applications. This work shows interesting results and adds important contributions such as the conversion of chicken feet, by-products in the poultry industry with low value in Brazil, to new material with high quality, such as the gelatin and films. Additionally, the data obtained provide important scientific information for food, heath, pharmaceuticals, and other areas. Therefore, chicken feet merit special attention because they appear to be a good alternative source for material and high-quality products.

## Figures and Tables

**Table 1:** Composition of gelatins produced in this study.

Contents (%)	Chicken Gelatin	Commercial Gelatin
Protein	70.9	18
Sugar	28.4	72
Citric acid	0.2	ND *
Salt	0.01	0.3
Colorant	0.1	ND
Flavor	0.4	ND
Microorganism (UFC/100 mL)	0	0
Pathogenic M. (UFC/100 mL)	0	0

* ND = not disclosed by manufacturer. UFC: colony forming unit

**Table 2:** Formulation of bio-therapeutic film of collagen from chicken.

Formulation I (with Glycerin)	Formulation II (with Propylene Glycol)
Collagen from chicken 10%	Collagen from chicken 10%
Glycerin 5%	Propylene glycol 5%
Methylparaben 0.1%	Methylparaben 0.1%
Distilled water 100 mL	Distilled water 100 mL

**Table 3:** Formulation of biotherapeutic with collagen from chicken.

Formulation III (with Glycerin)	Formulation IV (with Propyleneglycol)
Gelatin from swine 10%	Gelatin from swine 10%
Glicerin 5%	Propyleneglicol 5%
Methilparaben 0.1%	Methilparaben 0.1%
Distilled water 100 mL	Distilled water 100 mL

**Table 4:** Experimental design and the results of the collagen extraction process.

Assay	*x*_1_	*x*_2_	*x*_3_	[*A*_c_] (%)	*t* (h)	*T* (°C)	*Y*_exp_ (%)	*Y*_calc_ (%)
1	−1	−1	−1	1	3	50	4.4384	4.2493
2	1	−1	−1	3	3	50	5.1980	5.1505
3	−1	1	−1	1	7	50	1.6962	1.8785
4	1	1	−1	3	7	50	5.1659	5.4897
5	−1	−1	1	1	3	70	8.5713	8.3821
6	1	−1	1	3	3	70	8.1620	8.1145
7	−1	1	1	1	7	70	2.8835	3.0657
8	1	1	1	3	7	70	6.1218	6.4457
9	0	0	0	2	5	60	6.2332	6.0421
10	0	0	0	2	5	60	6.2700	6.0421
11	0	0	0	2	5	60	5.5907	6.0421
12	1.682	0	0	3.682	5	60	7.6714	7.4079
13	−1.682	0	0	0.318	5	60	4.1287	4.2016
14	0	1.682	0	2	8.364	60	2.5070	1.9702
15	0	−1.682	0	2	1.034	60	5.4152	5.7617
16	0	0	1.682	2	5	76.82	6.1483	6.0530
17	0	0	−1.682	2	5	43.18	7.0203	6.9252

where: [*A*_c_] = acetic acid concentration, *t* is time, *T* = temperature, and *Y* = extraction yield.

**Table 5:** Evaluation of model fit by variance analysis (ANOVA) at a 95% confidence level.

Source	Square Sum	Degree Freedom	Square Mean	*F*_calc_
Regression	57.832	11	5.257	
Residual	1.146	5	0.229	22.947
Fitting fault	0.854	3	0.285	
Error	0.292	2	0.146	1.950
Total	58.977	16		
% explaining variance				98.059
% maximum explanable variance				99.505
Coefficient of determination (*R^2^*)				0.9806

***F_tab1_***_(95%. 11, 5)_ = 4.070 and ***F_tab2_***_(95%. 3, 2)_ = 19.160.

**Table 6:** Chemical composition of chicken feet and their powdered collagen.

Composition *	Chicken Feet (g/100 g)	Powdered Collagen (g/100 g)
Moisture	60.009	9.749
Ashes	9.943	4.807
Lipids	12.875	6.919
Proteins	17.173	78.525

* Average of three batches under optimal extraction conditions.

**Table 7:** Gel strength analysis of collagen from chicken feet.

Gelatin	Collagen Contents (*w/w*%)	Gel Strength (kPa) *
Chicken feet	1.00	19.87
	2.00	44.65
	3.33	96.47
	4.00	119.1
	6.67	294.79
	10.0	466.87
Commercial	6.67	204.05

* Average of three batches under optimal extraction conditions.

**Table 8:** Results of ICP-MS analysis on powdered collagen from chicken feet.

Elements	Ash Content (µg/g) *	Total in Collagen (mg/100 g)
Li	0.050	2.10^−4^
Be	0.090	4.10^−4^
B	0.050	2.10^−4^
Na	5938	28.55
Mg	1834	8.817
Al	10.44	0.050
Si	22.53	0.108
P	168.1	0.808
S	1505	7.236
K	853.9	4.105
Ca	1692	8.136
Ti	2.060	0.010
V	0.010	5.10^−5^
Cr	<dl	-
Mn	0.100	5.10^−4^
Fe	3.210	0.015
Co	<dl.	-
Ni	0.150	7.10^−4^
Cu	1.350	0.006
Zn	<dl	-
Br	5.700	0.027
Sr	3.810	0.018
Ag	1.300	0.006
Cd	<dl	-
Sn	0.140	6.10^−4^
Ba	1.970	0.009
Pb	14.54	0.070

<d.l. = below detection limit. * average of 6 analyses of three batches under optimal extraction conditions.

**Table 9:** Sensory comparison of chicken and commercial gelatins at a 95% confidence level.

Flavour	Sample	Consumption Intention (%)	Attribute Evaluation *				
			Aroma	Appearance	Flavor	Texture	Overall
Pineapple	Commercial	94%	7.0 ± 1.1 ^a^	7.1 ± 1.8 ^b^	7.1 ± 1.6 ^c^	6.7 ± 1.7 ^d^	7.0 ± 1.6 ^e^
	Experimental	83%	7.1 ± 1.9 ^a^	7.5 ± 2.4 ^b^	6.3 ± 2.8 ^c^	6.5 ± 2.4 ^d^	6.8 ± 2.4 ^e^
Grape	Commercial	88%	8.3 ± 0.9 ^f^	8.1 ± 0.7 ^h^	8.3 ± 0.6 ^i^	7.9 ± 0.9 ^k^	8.2 ± 0.8 ^l^
	Experimental	83%	7.5 ± 1.6 ^g^	7.7 ± 1.5 ^h^	6.9 ± 2.5 ^j^	6.3 ± 2.4 ^k^	7.1 ± 2.0 ^m^

* similar letter indicates that there is no difference between the samples; *t*_tabled, 95%_ = 1.66.

**Table 10:** Comparative organoleptic properties of biotherapeutic films obtained from chicken feet using propylene glycol and glycerin.

Sample	pH	Color	Texture	Odor
Biotherapeutic colllagen films from chicken feet with glycerine	5.72	Dark yellow	Malleable, soft, slightly sticky touch but, with soft touch	Characteristic
Biotherapeutic colllagen films from chicken feet with propylene glycol	5.78	Dark yellow	Malleable, soft, slightly sticky touch but, with rough touch	Characteristic

**Table 11:** Comparative organoleptic characteristic of biotherapeutic collagen films from swine.

Sample	pH	Color	Texture	Odor
Biotherapeutic colllagen films from swine with glycerine	5.63	Light yellow Almost transparent	Malleable, soft, dry and soft touch	Odorless
Biotherapeutic colllagen films from swine with propylene glycol	5.71	Light yellow Almost transparent	Malleable, soft, dry and rough touch	Odorless
